# Migratory Status Is Not Related to the Susceptibility to HPAIV H5N1 in an Insectivorous Passerine Species

**DOI:** 10.1371/journal.pone.0006170

**Published:** 2009-07-09

**Authors:** Donata Kalthoff, Angele Breithaupt, Barbara Helm, Jens P. Teifke, Martin Beer

**Affiliations:** 1 Institute of Diagnostic Virology, Friedrich-Loeffler-Institut, Greifswald-Insel Riems, Germany; 2 Institute of Infectology, Friedrich-Loeffler-Institut, Greifswald-Insel Riems, Germany; 3 Max Planck Institute for Ornithology Seewiesen and Andechs, Andechs, Germany; U.S. Naval Medical Research Center Detachment/Centers for Disease Control, United States of America

## Abstract

Migratory birds have evolved elaborate physiological adaptations to travelling, the implications for their susceptibility to avian influenza are however unknown. Three groups of stonechats (*Saxicola torquata*) from (I) strongly migrating, (II) weakly migrating and (III) non-migrating populations were experimentally infected with HPAIV H5N1. The different bird groups of this insectivorous passerine species were infected in autumn, when the migrating populations clearly exhibit migratory restlessness. Following infection, all animals succumbed to the disease from 3 through 7 days post inoculation. Viral shedding, antigen distribution in tissues, and survival time did not differ between the three populations. However, notably, endothelial tropism of the HPAIV infection was exclusively seen in the group of resident birds. In conclusion, our data document for the first time the high susceptibility of an insectivorous passerine species to H5N1 infection, and the epidemiological role of these passerine birds is probably limited due to their high sensitivity to HPAIV H5N1 infection. Despite pronounced inherited differences in migratory status, the groups were generally indistinguishable in their susceptibility, survival time, clinical symptoms and viral shedding. Nevertheless, the migratory status partly influenced pathogenesis in the way of viral tropism.

## Introduction

Influenza A viruses are classified on the basis of two proteins expressed on the surface of virus particles; the hemagglutinin (HA) and neuraminidase (NA) glycoproteins [1]. To date 16 different HAs and nine different NAs are known and wild birds are reservoir hosts for all subtypes of avian influenza viruses [2]–[4].

Avian influenza viruses can be categorized on the basis of the clinical symptoms they cause in gallinaceous birds. Low pathogenic avian influenza (LPAI) caused by viruses belonging to all known hemagglutinins induce subclinical infections as well as a range of mainly respiratory and enteric symptoms. In contrast, highly pathogenic avian influenza (HPAI) viruses cause high mortality rates in gallinaceous poultry [5]. HPAI viruses have been restricted to subtypes H5 and H7, though not all viruses of these subtypes are highly pathogenic. Prior to the HPAI H5N1 virus epidemics in 2002, wild bird mortality associated with AI virus infection had been rare, consisting of sporadic cases usually spatially associated with HPAI virus outbreaks in domestic poultry [6], [7].

In contrast, HPAIV H5N1 Asia is unusual in that it has demonstrated high mortality rates in several outbreaks in wild birds [8]–[10], and the subsequent spread of these viruses to Europe and Africa suggests that the mid- to long-range transfer of these viruses may also have occurred through migratory birds [10]–[13]. Passerine birds have been naturally affected by HPAI H5N1 viruses [summarized at www.nwhc.usgs.gov/disease_information/avian_influenza/affected_species_chart.jsp, [14]–[17]]. Experimental infections of passerine species, e.g. house sparrows, European starling [18], [19], zebra and house finches [19] also characterized these birds as vulnerable, but their susceptibility differed as a function of the virus isolate used. Additionally, Gronesova et al., 2008 found that 18% of samples from 12 passeriform species tested positive for influenza A viral genome in a surveillance study conducted with 105 individuals [20]. Whether these birds might contribute to viral spread and must be considered in epidemiological evaluations is debatable. So far, no passerine birds could be detected as H5N1 infected during the large outbreaks among hundreds of monitored wild birds in Germany in 2006 and 2007 [10]. Nevertheless for the infection of several domestic cats during this outbreak episode [21], passerine birds were discussed as the most likely source of infection. The most probable reason for this phenomenon are the difficulties in finding sick or dead wild birds in the environment after mortality events, particularly those of small body size [22]. For example, in a study carried out to simulate mortality of ducks by lead poisoning and avian cholera, carcasses of ducks were spread over an area which was then intensely and systematically searched with the result that only 6% of the bodies were found [23].

The role of migratory birds in transferring the HPAIV H5N1 over long distances is a matter of controversial discussion, but needs to be assessed for predicting viral spread [24]–[27]. A potentially crucial, but so far almost unaddressed factor for understanding the role of migratory birds, are wide-spread physiological adaptations for their journeys [28]. The stunning performance of avian migrants, for example in non-stop flights across oceans [29], is made possible by a suite of specializations [28]. Migrants differ from residents in some permanent traits, such as morphology, but also by undergoing extensive seasonal changes in physiology in preparation for migration [28], [30]. These changes, based on inherited programs, include major adjustments of organs and metabolism, and may also extend to the immune system [28], [31]. Possible implications for the susceptibility of wild birds are open since the migratory status can cause both immuno-enhancing and immuno-suppressive specializations to enable maximal performance [28], [31].

We had the unique opportunity to investigate the susceptibility of a passerine species, the stonechat (*Saxicola torquata*), to a recent H5N1 clade 2.2 isolate in relation to the migratory status. Stonechats are small, insectivorous songbirds that differ widely in inherited migratory programs [30]. Three different populations were included: (I) obligatory, strong European and West Siberian migrants, (II) weak, partial European migrants, and (III) African residents [30], [32]. All birds were infected with a recent HPAIV H5N1 isolate to determine whether the migratoriness affects susceptibility in the same species. Birds were infected during migration season to examine whether the migratory status influences the immune capacity against HPAIV infection and whether HPAI H5N1-infected birds would still be able to migrate.

## Materials and Methods

### Trial approval

The trial was evaluated by the responsible ethics committee of the State Office for Agriculture, Food Safety and Fishery in Mecklenburg-Western Pomerania (LALFF M-V) and gained governmental approval under the registration number LVL M-V/TSD/7221.3-1.1-003/07.

### Virus propagation

For the infection experiments, the 3^rd^ passage of the well-defined strain A/*Cygnus cygnus*/Germany/R65/2006 (H5N1) originating from a dead whooper swan found in early February 2006 on the island of Ruegen, was used [33]–[35]. Allantoic fluid from inoculated embryonated hens eggs was collected and stored at −70°C until usage.

All experiments using HPAI H5N1 virus were conducted using biosaftey level 3 agriculture containment procedures at the Friedrich-Loeffler-Institut (FLI), Insel Riems.

### Animals and experimental design

26 adult stonechats (*Saxicola torquata*) were provided by the Max Planck-Institute for Ornithology, Seewiesen and Andechs, Germany. At the time of the experiments, the birds were between 2 and 9 years old (mean 3.65, StD 1.55 yrs). Most birds hatched in captivity, typically as F1 offspring from wild-derived birds. 1 African resident, 1 Irish weak migrant, and 2 European strong migrants were collected in the wild as hatchlings. Migratory behaviour of captive stonechats in the lab is robust and persists over many years, even under constant light, food, and temperature conditions [36]. Ten birds represented strongly migrating populations from Austria and Kazakhstan (group I), ten individuals belonged to a weakly and partially migrating population from the British Isles (group II), and six birds originated from a non-migrating population from equatorial Kenya (group III) [30], [32]. Although no physiological data exist from the time directly prior to experimentation, there were no differences in selection criteria for groups I, II, and III, nor were there differences in age or sex composition. Experimental infection of the stonechats was conducted in October, when stonechats exhibit their characteristic migratory restlessness (‘Zugunruhe’) that indicates migratory status.

The animals were housed in individual cages in the high containment facility of the FLI with a 12-hour lighting regimen. Feed and water were provided *ad libitum*. All three groups of stonechats were inoculated oculo-oronasally with a 50% egg infectious dose (EID_50_) of 10^6.0^ HPAIV H5N1 per animal in 0.25 ml of cell culture medium. Within each group, one bird was mock-inoculated and housed within the same stable unit in a separate cage at a distance of approximately 10 cm to infected birds.

All birds were monitored daily for clinical signs. Oropharyngeal swabs were collected every three to four days and samples from individual feces/cloaca were collected almost daily for 21 days in Dulbecco's modified Eagle medium (DMEM), supplemented with 5% fetal bovine serum (FBS) and antimicrobial drugs (enrofloxacin 1 mg/ml, gentamicin 0.05 mg/ml, lincomycin 1 mg/ml). If an individual bird exhibited severe symptoms, it was humanely killed.

### Virus isolation and real-time RT-PCR

All swabs were stored at −70°C until virus detection was performed. All individual samples were tested with real-time-RT-PCR (rRT-PCR) specific for H5, and the genomic load was semi-quantified [37]. Viral titers of the oropharyngeal and cloacal/fecal swab samples were calculated as 50% tissue culture infectious dose (TCID_50_) per ml swab sample on Madin-Darby canine kidney (MDCK) cells (Collection of cell lines in veterinary medicine, FLI Island of Riems, RIE1061).

### Serology

Pre-experimentally, sera were collected from all birds. The serum samples were heat inactivated at 56°C for 30 min and examined for the presence of antibodies against the nucleoprotein of avian influenza virus type A using a commercially available competitive ELISA kit (Pourquier AI A Blocking ELISA; Institut Pourquier, Montpellier, France).

### Gross pathology, histopathology, and immunohistochemistry

From all birds, tissues of trachea, lungs, heart, cerebrum, cerebellum, spinal cord, proventriculus, gizzard, small and large intestine, liver, pancreas, spleen, skin, kidney, adrenal glands, cranium and femur were sampled. Subsequent tissues were formalin-fixed and processed for paraffin-wax embedding according to standardized procedures. As earlier described [38] immunohistochemistry for influenza virus A nucleoprotein (NP) was performed. Briefly, after dewaxing sections were microwave-irradiated for antigen retrieval (2×5 min, 600 W, 10 mM citrate buffer pH 6.0), and were incubated with a rabbit anti-NP serum (1∶750). A biotinylated goat anti-rabbit IgG1 (Vector, Burlingame, CA, USA) was applied (1∶200) as secondary antibody. A bright red intracytoplasmic and nuclear signal was observed by means of the avidin-biotin-peroxidase complex method. Positive control tissues of chickens experimentally infected with HPAI virus (H5N1) and, additionally, a control primary rabbit serum against bovine papillomavirus (BPV 1∶2000) was included.

### Statistical evaluation

Results were statistically evaluated by Fisher-Freeman-Halton's test to verify the association of unordered r x c tables (level of significance alpha 0.05).

## Results

**Figure 1:**
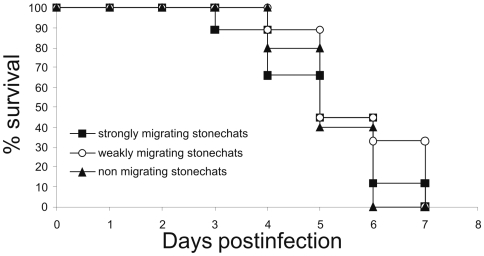
Mortality of stonechats with different migration patterns after inoculation with A/*Cygnus cygnus*/germany/R65/2006 H5N1 virus. Percent survival of strongly migrating versus weakly migrating versus resident stonechats inoculated with 10^6^ EID_50_/animal expressed as mean value of individuals per group.

All pre-experimental sera from the individual birds tested negative for antibodies against the nucleoprotein of influenza A virus. After infection, for both migratory groups and the resident stonechats, similar incubation periods of 3 to 4 days could be observed, and all groups succumbed to the disease from 3 through 7 days post infection (DPI; Figure 1). Most animals died acutely without developing any visible clinical signs. Only a few birds exhibited detectable neurological disorders, such as severe ataxia and torticollis. In contrast, all non-inoculated animals remained healthy.

**Figure 2:**
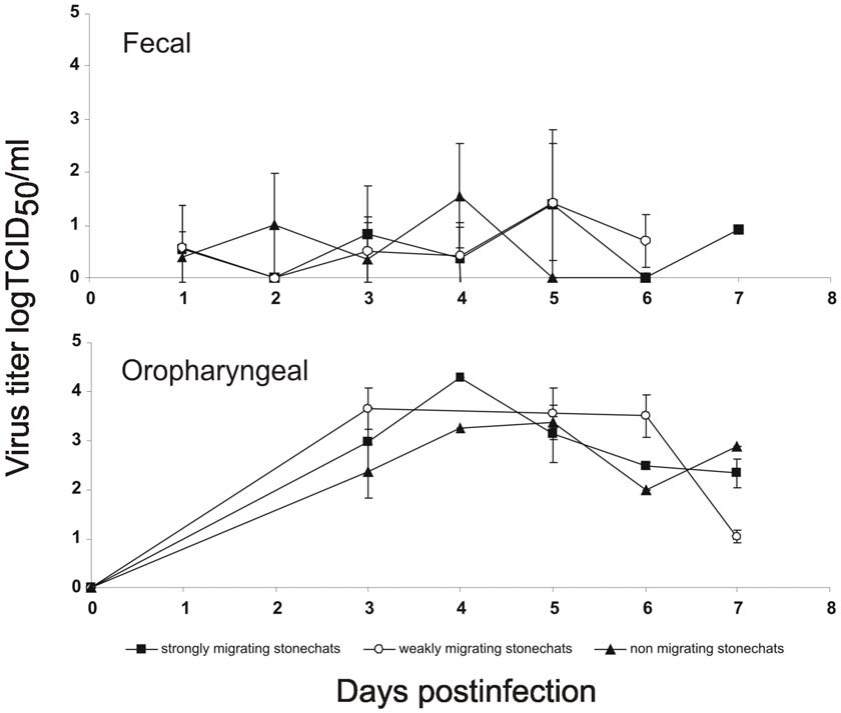
Viral shedding pattern from passerine stonechats inoculated with HPAIV H5N1. Titres of replicating virus from fecal and oropharyngeal swab samples expressed as TCID_50_/ml. Note that groups of birds were not tested every other day (for oropharyngeal swab samples) and number of individuals per group is differing. Standard deviations are shown as error bars.

HPAIV H5N1 shedding in oropharyngeal and fecal/cloacal swabs was monitored daily by virus titration on MDCK cells (Figure 2). Both migratory groups (Figure 2) and the resident stonechats excreted virus from 1 through 7 DPI in fecal samples, and likewise, shedding from oropharyngeal samples was demonstrated from 3 through 7 DPI (Figure 2). Furthermore, in all populations rRT-PCR revealed positive viral genome for the same period, whereas all swab samples taken from control birds remained negative (data not shown). Generally, viral titres in the oropharyngeal swabs were slightly higher than titres detected in fecal samples (Figure 2).

Semi-quantified viral RNA loads of different organs and tissues are summarized in table 1. Viral loads are expressed as cycle of threshold value (ct), and the more genome is detected the lower ct-values are given. Organs from the tested stonechats showed a wide range of RNA loads with pancreas, myocardium, CNS and adrenals consistently exhibiting the highest copy numbers in all three populations (table 1).

**Table 1:** Distribution of viral genomic load† and influenza A antigen in tissues of stonechats after challenge infection with HPAIV H5N1.

Organ	IHC birds positive/birds inoculated			IHC pos/	Celltype affected	Histopathology
	Viral RNA load in tissue pos/total(ct-value min−max†)					
	Strongly	Weakly	Non	Total		
	migrating	migrating	migrating	%		
Pancreas	9/9	8/9	5/5	22/23	Exocrine epithelium	Necrosis, mild pancreatitis
	9/9 (15.2–23)	9/9 (15.8–26)	5/5 (14.5–22.3)	96%	Endocrine epithelium	
Heart	9/9	7/9	5/5	21/23	Cardial myocytes	Myocardial degeneration, mild myocarditis
	9/9 (19–30.9)	9/9 (17.1–25)	5/5 (20.5–28)	91%		
CNS	7/9	7/9	5/5	19/23	Neurons, glial cells	Neuronal necrosis, Neuronal degeneration
	9/9 (20.9–30.6)	9/9 (22.2–31.2)	5/5 (22–32.9)	83%	Ependymal cells	Neuronophagia, glia nodules
Adrenal	5/6	6/7	2/3	13/16	Cortical epithelium	Necrosis, moderate adrenalitis
	9/9 (19.3–25)	9/9 (18.2–27.5)	5/5 (19.8–25.1)	81%	Medullar epithelium	
Lung	7/9	5/9	5/5	17/23	Pneumocytes	Edema, congestion, epithelial necrosis
	8/9 (20.7–32)	9/9 (20.9–32)	5/5 (19.8–32)	74%	(Para-) bronchial epithelium	Epithelial degeneration, mild pneumonia
Pecten oculi	5/7	3/7	2/3	10/17	Endothelium	no lesion
				59%		
Trachea	6/8	2/9	2/3	10/20	Epithelium	Necrosis, Epithelial proliferation
	9/9 (22.2–33.5)	9/9 (20.3–29.6)	5/5 (19.1–27)	50%		Epithelial degeneration, moderate tracheitis
Kidney	1/9	2/9	3/5	6/23	Tubular epithelium,	no lesion
	9/9 (25.3–30.6)	9/9 (23.3–31.8)	5/5 (20.5–30.6)	26%	Ganglia	
Liver	1/9	0/9	3/5	4/23	Hepatocytes	Hepatocyte degeneration, mild heapatitis
	9/9 (21.2–32.3)	9/9 (24.5–32.7)	5/5 (22.2–30.9)	17%		
Intestine	3/9	0/9	2/5	5/23	Mucosal epithelium	no lesion
	9/9 (23.1–33.3)	9/9 (19.7–35.4)	4/5 (19.8–31)	22%		
Gizzard	1/9	0/9	0/5	1/23	Smooth muscle cells	no lesion
	9/9 (24.7–31.2)	8/8 (19.3–30.5)	5/5 (21.4–31.2)	4%		
Skin	0/9	0/9	1/5	1/23	Feather follicle	no lesion
				4%	epidermal epithelium	
Nose	2/3	1/1	5/5	8/9	Respiratory epithelium	Necrosis, moderate rhinitis
	8/9 (19.3–27.8)	8/9 (18.7–30.3)	5/5 (18.8–27.2)	89%	Glandular epithelium	
Harderian Gl.	5/6	6/6	3/3	14/15	Glandular epithelium	Necrosis
				93%		
Spleen	0/3	0/6	0/4	0/13		Severe depletion
	9/9 (21.5–29.7)	8/8 (21.9–29.3)	5/5 (21–28.8)	0%		
Endotheliotropism*	0/9	0/9	3/5	**3/23**		
				**13%**		
Epitheliotropism	9/9	8/9	5/5	**22/23**		
				**96%**		
Neurotropism	9/9	9/9	5/5	**23/23**		
				**100%**		

† Viral RNA detected by real-time reverse transcription-PCR (rRT-PCR) in birds after challenge infection with highly pathogenic avian influenza virus strain A/Cygnus cygnus/Germany/R65/06 (H5N1). Real-time RT-PCR results are presented as cycle of threshold (Ct)-values: >35 scored as negative.

* Statistical significant (alpha 0.05) difference between non-migrating and migrating populations according to association of unordered r x c tables by Fisher-Freeman-Halton's test.

### Gross pathology

**Figure 3:**
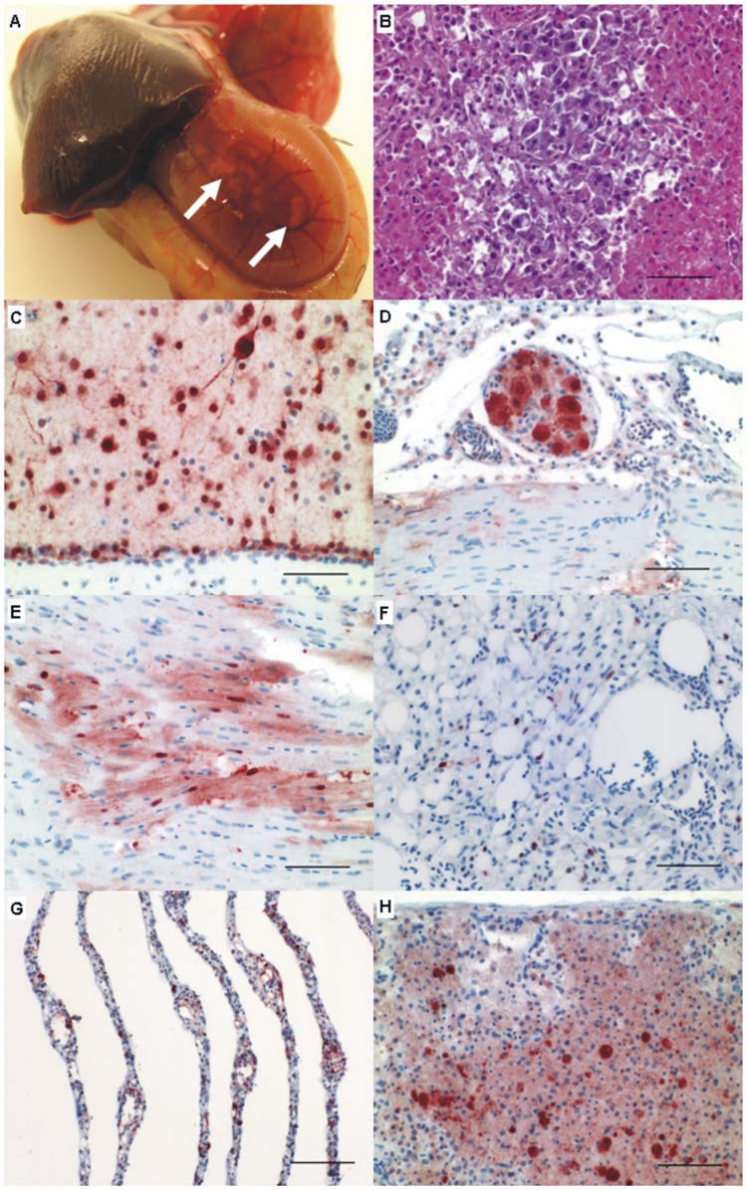
Gross pathology, histopathology and immunohistochemistry for nucleoprotein of avian influenza virus. (A) Pancreas; weakly migrating stonechat at 7 DPI. Multifocal to coalescent necrosis (arrows). (B) Pancreas; weakly migrating stonechat at 7 DPI. Focally extensive vacuolar degeneration and necrosis of pancreatic parenchyma. HE. Bar 50 µm. (C–H) Immunohistochemistry. ABC method, hematoxylin counterstain. (C) Brain; weakly migrating stonechat at 5 DPI. Intranuclear and intracytoplasmic staining in neurons, glial and ependymal cells. Bar 50 µm (D) Heart; weakly migrating stonechat at 5 DPI. Intense immunostaining in extracardial ganglion cells of the peripheral nervous system. Bar 50 µm (E) Heart; weakly migrating stonechat at 5 DPI. AIV antigen staining within degenerating cardiomyocytes. Bar 50 µm (F) Lungs; strongly migrating stonechat at 3 DPI. AIV antigen in scattered pneumocytes. Bar 50 µm (G) Pecten oculi, non-migrating stonechat at 5 DPI. Influenza virus antigen detected in endothelial cells. Bar 100 µm (H) Harderian gland; weakly migrating stonechat at 6 DPI. Widespread acute coagulative necrosis of the glandular acini with intralesional AIV antigen. Bar 50 µm.

At necropsy between 3 and 7 DPI, the predominant findings in all birds were mild to moderate edema and congestion of the lungs. Four stonechats showed multiple sharply demarcated white foci of up to two millimeters in diameter in the pancreas, and the surrounding parenchyma was blurred grey-red (Figure 3a).

### Histopathology and immunohistochemistry (Table 1, Figure 3b–h)

Histomorphological investigations in principle did not reveal differences in the lesions or staining patterns between strongly (group I), weakly (group II) or non-migrating (group III) stonechats.

Influenza virus antigen was frequently found within the cytoplasm and nucleus of ***pancreatic acini*** (22/23 = 96%: 9/9 I, 8/9 II, 5/5 III;) in coalescent foci of coagulative necrosis (figure 3b), and in ***cardiomyocytes*** (21/23 = 91%: 9/9 group I, 7/9 group II, 5/5 group III) with vacuolar degeneration accompanied by mild myocarditis (Figure 3e). In addition, in the ***central nervous system, CNS*** (19/23 = 83%: 7/9 I, 7/9 II, 5/5 III) there was staining in neurons, glial and ependymal cells (Figure 3c) associated with neuronal necrosis, neuronophagia and vacuolization of the neuropil ( = edema). Viral antigen was also present in neurons of the peripheral nervous system (ganglia, Figure 3d). In the **lungs**, influenza antigen was detected in pneumocytes, bronchial and parabronchial epithelium (17/23 = 74%: 7/9 I, 5/9 II, 5/5 III), with epithelial degeneration, necrosis, and mild pneumonia (Figure 3f). The lungs were congested and edematous. In contrast to the lungs, viral antigen in tracheal epithelium was rarely observed (10/20 = 50%, 6/8 I, 2/9 II, 2/3 III).

The ***Harderian gland*** (14/15 = 93%:5/6 I, 6/6 II, 3/3 III), and the ***nasal respiratory epithelium*** (8/9 = 89%:2/3 I, 1/1 II, 5/5 III) were both affected in almost every tested stonechat and necrotic epithelium stained strongly positive (Figure 3h). Regarding the ***adrenal gland***, the interrenal cells ( = cortical cells) were more intensely affected compared to the chromaffin cells ( = medullary cells), with widespread cortical necrosis and mild mixed cellular infiltrates (13/16 = 81%: 5/6 I, 6/7 II, 2/3 III).

In less than 30% of the investigated animals, influenza virus antigen was detected in the tubular epithelium of the ***kidney*** (6/23 = 26%: 1/9 I, 2/9 II, 3/5 III) and within the ***liver*** in the hepatocytes (4/23 = 17%: 1/9 I, 0/9 II, 3/5 III). Sporadically we found antigen within feather follicles in the skin and smooth muscle cells in the gizzard.

In particular ***endotheliotropism*** (3/23 = 13%: 0/9 I, 0/9 II, 3/5 III) was variable between the groups. Statistical evaluation to test the association of unordered r x c tables by Fisher-Freeman-Halton's test revealed that the observed difference of endotheliotropism of HPAIV between the groups was significant (alpha 0.05). In numerous birds we found antigen staining in endothelial cells of the ***pecten oculi*** only (10/17 = 59%; Figure 3g). Two stonechats showed viral antigen restricted to endothelial cells of the pecten oculi and the heart. Because of the restricted distribution these findings were not classified as “true” endotheliotropism, while three further birds exhibited a widespread endotheliotropism. The liver, lung, kidney, gizzard, intestine, heart and pecten oculi were typically affected organs. Both epitheliotropism and neurotropism were detected in all three populations and statistical analyses revealed no significant differences.

All samples of control animals as well as bone, esophagus, and the skeletal musculature of infected birds stained negative and did not reveal any histologic lesions.

## Discussion

The present study examined whether migratory status and associated physiological specializations affect the response of a songbird species to infection with HPAIV H5N1. Migratory performance is associated with a suite of adaptations that include preparatory, seasonal modification of body composition and metabolism [28], [29]. Such recurring preparations for migration are driven in many passerines, including stonechats, by inherited programs and occur even in the absence of environmental influences [30], [39]. Adjustments of physiology are likely to also affect the immune system and could lead to either temporary down-regulation [31] or up-regulation of immune functions. It is for example known that unspecific stress induced by injection of lipopolysaccharide caused less symptoms in migratory than in resident stonechats (B. Helm unpublished data), raising the question whether this is also the case after specific immunological exercise.

All inoculated individuals shed virus in respiratory secretions and feces; shedding generally increased with time and reached a maximum within 3 to 6 DPI. Migratory and non-migratory stonechats could not be discriminated on the basis of clinical symptoms or virus shedding patterns. Histomorphologically, there was neither a difference in the staining pattern nor in the severity of damage and degree of immunostaining in the affected tissues, and a marked neuro- and epitheliotropism was detected in all three populations. The affection of the ocular endothelium and the respiratory nasal epithelium was likely a consequence of the oculo-oronasal infection route. Due to our data we hypothesize that the infection of the nasal epithelium and ocular endothelium led to viremia, followed by viral spreading and manifestation mainly in the pancreas, heart, CNS and lung. Although there was no indication, the infection of the CNS via an ascending neuronal pathway should not be excluded [40]. In accordance with published data [19] the staining pattern in birds belonging to the order Passeriformes varies, and neurotropism seems to play a central role for the rapid course of disease. Besides this, endotheliotropism was prevalent in the non-migrating population, and the widespread tropism led to high viral RNA loads in a broad range of organs, but was not directly associated with survival time. Endotheliotropism is rather common in H5N1 HPAIV infected chicken, and is occasionally observed in other avian species such as swans [34], [41], [42] and other passerine birds [19]. However, endotheliotropism is not strictly correlated with early death in these reports.

Interestingly, the non-migrating population of stonechats significantly more often showed positive influenza antigen staining in endothelia (Group I: 0/9; Group II: 0/9; Group III: 3/5). Although the number of individuals tested was limited in all groups, we can speculate that pathogenesis of HPAIV H5N1 infection may be modulated by the migratory status of an individual without influencing the final consequences of the infection. Whether this is an immunological function (e.g., unspecific immune stimulation) or somehow genetically determined is uncertain, but experimental infection of migratory stonechats in the stationary phase may provide this information in future studies. It may be that an activated metabolism during ‘Zugunruhe’ is beneficial, although it could not protect stonechats from succumbing to HPAI infection.

Several passerine species are known to be vulnerable, but experimental studies have focused on predominantly granivorous species that are easy to keep [18], [19]. Our data clearly show that also an insectivorous songbird species is susceptible. Furthermore, unique to this study compared to other published data concerning passerine species is the combination of immunohistochemistry findings with molecular virological data obtained by rRT-PCR. In order to define the tissue or cellular tropism, and to ensure that positive PCR results do not originate from viremia or high virus load in transudates in serous cavities, immunohistochemistry was performed. On the other hand, rRT-PCR is a more sensitive method useful to support antigen detection *in situ*. Limitations for successful histopathologic analysis of infection experiments with Passeriformes are small-sized samples, autolytic tissue or loosely arranged or singular areas of infected cells which are not targeted in every section. Therefore, a combined morphological and molecular approach seems to be beneficial for a deeper pathogenetic understanding of the mechanisms of the disease, and for comparative studies such as the present one.

In conclusion, our data document for the first time the high susceptibility of an insectivorous passerine species to H5N1 infection. The migratory status proved to be irrelevant for the outcome, which makes it unlikely that these birds play a role for virus transmission, especially for transfer over long distances. Nevertheless, taking into account the limitations with regard to sample size and the fact that just one songbird species was tested in this study, the migratory status influenced the pathogenesis concerning viral tropism, but this did not result in differences of survival time, clinical symptoms or viral shedding.
